# Clinical application of machine learning‐based pathomics signature of gastric atrophy

**DOI:** 10.3389/fonc.2024.1289265

**Published:** 2024-02-27

**Authors:** Yadi Lan, Bing Han, Tianyu Zhai, Qianqian Xu, Zhiwei Li, Mingyue Liu, Yining Xue, Hongwei Xu

**Affiliations:** ^1^ Department of Gastroenterology, Shandong Provincial Hospital, Shandong University, Jinan, Shandong, China; ^2^ Department of Gastroenterology, Shandong Provincial Hospital Affiliated to Shandong First Medical University, Jinan, Shandong, China

**Keywords:** pathology, machine learning, gastric, atrophy, cancer

## Abstract

**Background:**

The diagnosis of gastric atrophy is highly subjective, and we aimed to establish a model of gastric atrophy based on pathological features to improve diagnostic consistency.

**Methods:**

We retrospectively collected the HE-stained pathological slides of gastric biopsies and used CellProfiler software for image segmentation and feature extraction of ten representative images for each sample. Subsequently, we employed the Least absolute shrinkage and selection operator (LASSO) to select features and different machine learning (ML) algorithms to construct the diagnostic models for gastric atrophy.

**Results:**

We selected 289 gastric biopsy specimens for training, testing, and external validation. We extracted 464 pathological features and screened ten features by LASSO to establish the diagnostic model for moderate-to-severe atrophy. The range of area under the curve (AUC) for various machine learning algorithms was 0.835-1.000 in the training set, 0.786-0.949 in the testing set, and 0.689-0.818 in the external validation set. LR model had the highest AUC value, with 0.900 (95% CI: 0.852-0.947) in the training set, 0.901 (95% CI: 0.807-0.996) in the testing set, and 0.818 (95% CI: 0.714-0.923) in the external validation set. The atrophy pathological score based on the LR model was associated with endoscopic atrophy grading (Z=-2.478, P=0.013) and gastric cancer (GC) (OR=5.70, 95% CI: 2.63-12.33, P<0.001).

**Conclusion:**

The ML model based on pathological features could improve the diagnostic consistency of gastric atrophy, which is also associated with endoscopic atrophy grading and GC.

## 1 Introduction

According to the latest global cancer statistics, gastric cancer (GC) is one of the most common cancers and a significant contributor to cancer-related mortality due to late diagnosis (1). Intestinal gastric adenocarcinoma (GA) is the predominant type of GC, following a process known as the Correa cascade, progressing from chronic inflammation to atrophy and intestinal metaplasia, then to dysplasia, and finally to GC (2). Consequently, the early identification of atrophy and intestinal metaplasia plays a crucial role in the timely diagnosis and treatment of GC.

Biopsy remains the gold standard for diagnosis and staging of gastric atrophy. Pathologically, atrophy means a decrease in gastric intrinsic glands. According to the visual analog scale (VAS), it includes four grades: none, mild, moderate, and severe (3). Some researchers have developed the Operative Link on Gastritis Assessment (OLGA) (4) and Operative Link for Intestinal Metaplasia (OLGIM) (5) staging systems, which link pathological staging with cancer risk, indicating a significant increase in GC risk of stages III-IV. However, the pathological grading of atrophy is highly subjective, with low inter- and intra-observer agreement. Moreover, the diagnosis of atrophy has strict requirements for biopsy specimens. If endoscopists did not acquire the samples from muscularis mucosa, the pathologists could not identify the grade of atrophy.

Due to advances in slide scanning technology and the reduction of digital storage costs, the complete digitization of tissue slides has become possible. In recent years, the term pathomics has attracted increasing attention. Pathomics refers to capturing various data from digital pathology images to generate quantitative features, which are subsequently analyzed using different algorithms to determine diagnosis or predict survival outcomes (6, 7). This study aimed to extract digital pathological features from H&E stained pathological slides and use machine learning (ML) algorithms to construct diagnostic models for atrophic gastritis, which could improve diagnostic consistency and provide accurate real-time clinical decision support (CDS).

## 2 Methods

### 2.1 Study population

The Ethics Committee of Shandong Provincial Hospital (SWYX: NO.2023-031) approved the study. The study was retrospective, for which the Ethics Committee waived informed consent from patients.

**Figure 1:**
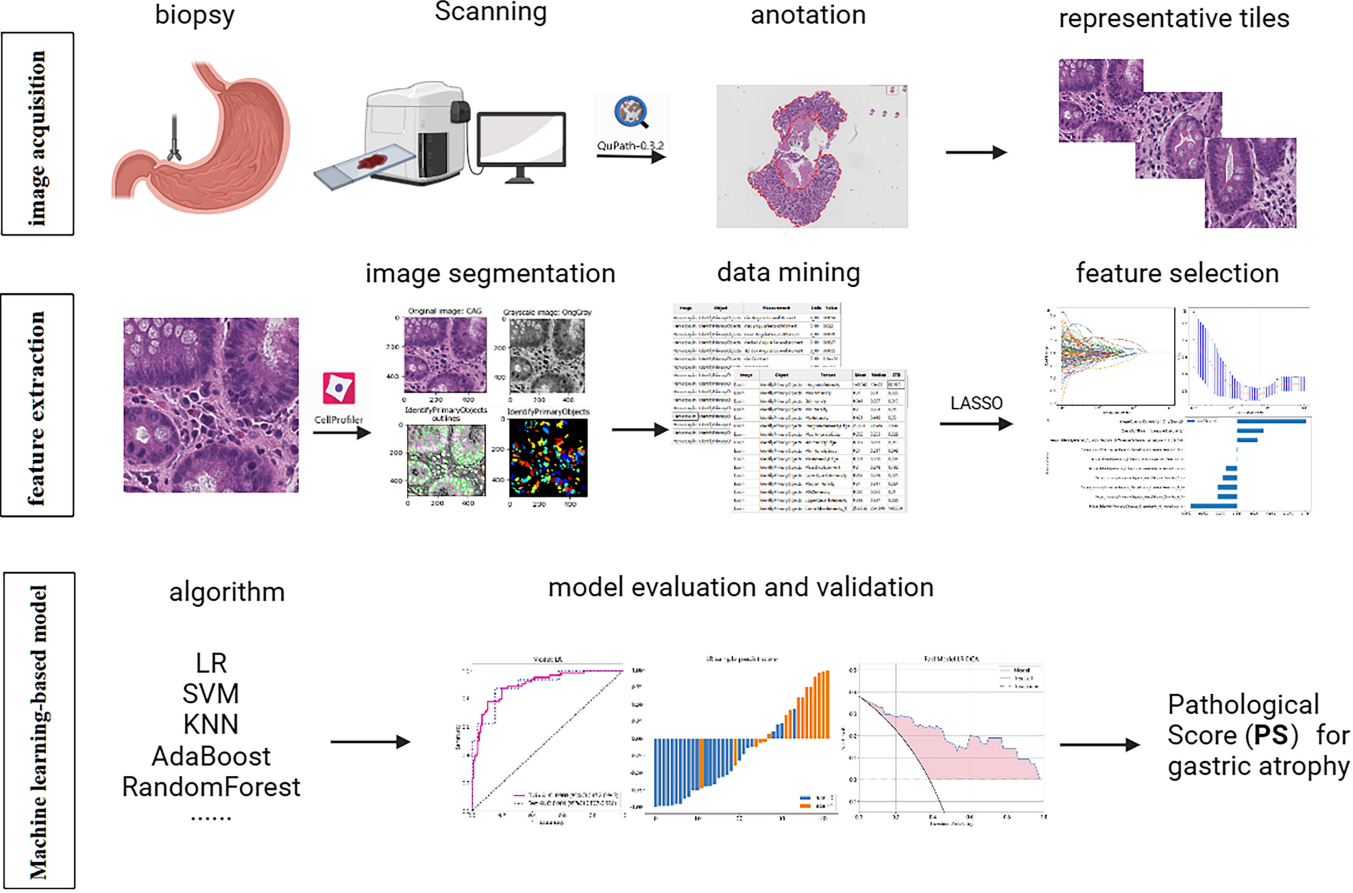
The workflow of the entire research design. Pathological features were extracted by Cellprofiler software and selected by LASSO. The diagnostic models were built by multiple machine learning algorithms and evaluated by AUC, histogram, and DCA. LASSO, Least absolute shrinkage and selection operator; LR, logistic regression; SVM, Support Vector Machine; KNN, K Nearest Neighbor; AUC, area under the curve; DCA, decision curve analysis.

We selected the pathological slides of patients in Shandong Provincial Hospital from January 2021 to June 2022 for model training and testing and October 2018 to December 2020 for external validation. Our study included patients who underwent gastric antrum biopsy for atrophy grading during gastroscopy with complete clinical information. We excluded patients whose pathological slides were difficult to obtain, were poorly stained, or lacked muscularis mucosa. **Figure 1** presents the overall flow of the study.

### 2.2 Acquisition of images

We scanned the Hematoxylin-eosin-stained (H&E-stained) slides prepared by formalin-fixed paraffin-embedded samples at 40x and saved them in svs format. Two gastrointestinal pathologists with more than ten years of experience independently assessed the degree of atrophy for each slide. The slides with disagreements performed a joint assessment conducted by two experts. Since there were several identical biopsies on an entire WSI, we used the Qupath software to annotate the most representative areas with the best staining in the WSI. The representative area referred to the mucosal glands without the white background. Regions demonstrating optimal staining exhibited superior transparency and a clear distinction between nuclear and cytoplasmic staining, devoid of artifacts (**Supplementary Figure S1**). Subsequently, we cropped the annotated areas in the WSI into patches with dimensions of 512x512 pixels and saved them as JPG files. Lastly, ten representative images, which exhibited the highest number of glands, were selected from the cropped images of each slide.

### 2.3 Extraction of pathological features

We constructed a pipeline (Document S1) for image segmentation and feature extraction using multiple modules in CellProfiler (8). We split the H&E stained image into hematoxylin and eosin stained images using the UnmixColors module (9) and converted to grayscale images using the ColorToGray module. Afterward, we employed the IdentifyPrimaryObjects module to segment the image and identify cell nuclei. Further, we utilized modules such as MeasureImageQuality, MeasureImageIntensity, MeasureColocalization, MeasureGranularity, MeasureTexture, MeasureObjectSizeShape, MeasureObjectIntensity, and MeasureObjectIntensityDistribution to extract quantitative pathological features. Finally, the extracted pathological features were exported and saved as an Excel file.

### 2.4 Feature selection and model construction

After normalizing the data, we calculated Pearson correlation coefficients between features. We would retain only one if the correlation coefficients between features were more than 0.9. We subsequently divided the data into training and validation sets with a ratio of 8:2. Further, we used the Least absolute shrinkage and selection operator (LASSO) regression to select non-zero coefficient features for subsequent analysis. LASSO achieves the effect of feature selection by reducing or even shrinking some regression coefficients to zero through the penalty term. After LASSO analysis, only features with a strong influence on the target variable will remain, while the coefficients of other features will become zero. As the penalty increases, the complexity of the model decreases, and the MSE (mean square error) increases. We aimed to find a suitable level of penalty to obtain fewer variables but still maintain a small MSE. In our study, only ten non-zero coefficient features remained after the LASSO. Finally, we constructed ML models using logistic regression (LR), Support Vector Machine (SVM), K Nearest Neighbor (KNN), Random Forests, ExtraTrees, extreme Gradient Boosting (XGBoost), Light Gradient Boosting Machine (LightGBM), GradientBoosting and AdaBoost.

### 2.5 Statistical analysis

We used the Student t-test to compare normally distributed continuous variables, the Chi-square test to compare categorical variables, and the non-parametric test to compare ordered variates or non-normally distributed continuous variables. Binary logistic regression (stepwise method) was further employed to estimate the adjusted odds ratio (OR) and 95% confidence interval (CI). We performed the Kappa test using the vcd package of R software and calculated 95% confidence intervals. When the kappa coefficient is less than 0.2, the consistency is poor; 0.21-0.40 is fair; 0.41-0.60 is moderate; 0.61-0.80 is strong; and 0.81-1.00 is very strong. We used the area under the curve (AUC) to assess the discrimination of the ML models, histograms to demonstrate the calibration of the models, and decision curve analysis (DCA) to evaluate the clinical applicability of the models. The AUC represents the area under the receiver operator characteristic (ROC) curve generated by the classifier based on different decision thresholds to assess the model performance. Overall, the model performance improves as the AUC approaches 1. Prediction histograms can display and analyze the distribution of prediction results, which helps us evaluate the model performance. The DCA is used to evaluate the net benefit of the model under different decision thresholds. We often draw a reference line, a baseline for making random decisions without any predictions. If the net benefit curve of the model is above the baseline, it indicates that the model has better clinical utility relative to the baseline. We used Statistical Package for Social Sciences (SPSS) v.24.0, R v.4.2.1, and Python v.3.1 to perform all analysis. P values <0.05 were considered significant.

## 3 Results

### 3.1 Data sets

We selected 289 pathological slides, including 169 for the model training, 42 for the testing, and 78 from different years for the external validation. Two professional pathologists independently diagnosed 289 slides, and the diagnostic agreement was strong - a Kappa value of 0.68 (95%CI: 0.60-0.77, P<0.001). The slides with disagreements performed a joint assessment conducted by two experts. Finally, the two pathologists reached a consensus on all the inconsistent slides by discussing them together.

### 3.2 Feature extraction and screening

**Figure 2:**
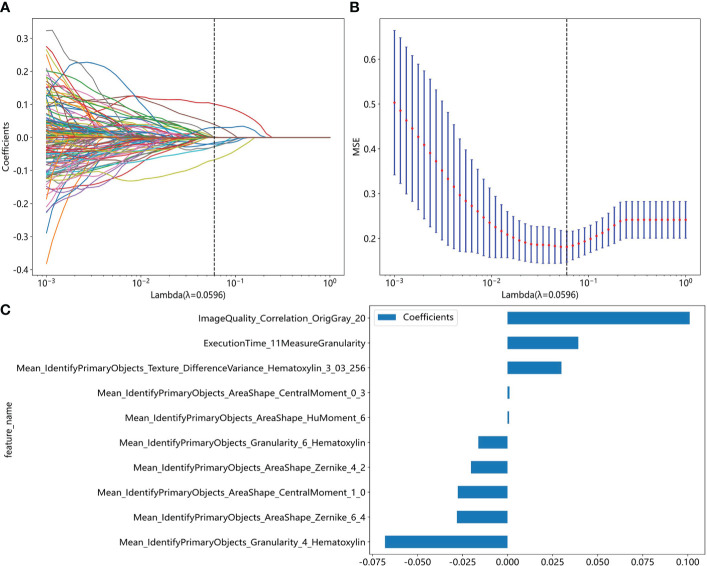
Pathomics feature selection based on the LASSO algorithm for moderate to severe gastric atrophy. **(A)** LASSO coefficient profiles of the features. The different color line shows the corresponding coefficient of each feature. As the penalty increases, Lasso regression adjusts the coefficient of some variables to zero, thus enabling variable selection. **(B)** Tuning parameter (λ) selection in the LASSO model. As the penalty increases, the complexity of the model decreases, and the MSE increases. We aimed to find a suitable level of penalty to obtain fewer variables but still maintain a small MSE. **(C)** Selected features weight coefficients. LASSO, Least absolute shrinkage and selection operator; MSE, mean square error.

We extracted 464 pathological features from 2890 images using Cellprofiler software. These features include 324 related to cell nuclei granularity, texture, size, shape, and pixel intensity distribution, 124 features related to image quality, intensity, co-localization, and correlation between intensities. We then calculated the average value of features extracted from 10 representative images for each slide. After removing 30 irrelevant or abnormal features, 434 remained (**Supplementary Table S1**), and we screened 203 features by Pearson correlation coefficient. Finally, we selected ten non-zero coefficient features by the Lasso regression (**Figure 2**).

### 3.3 ML-based diagnostic model for moderate to severe atrophy

**Figure 3:**
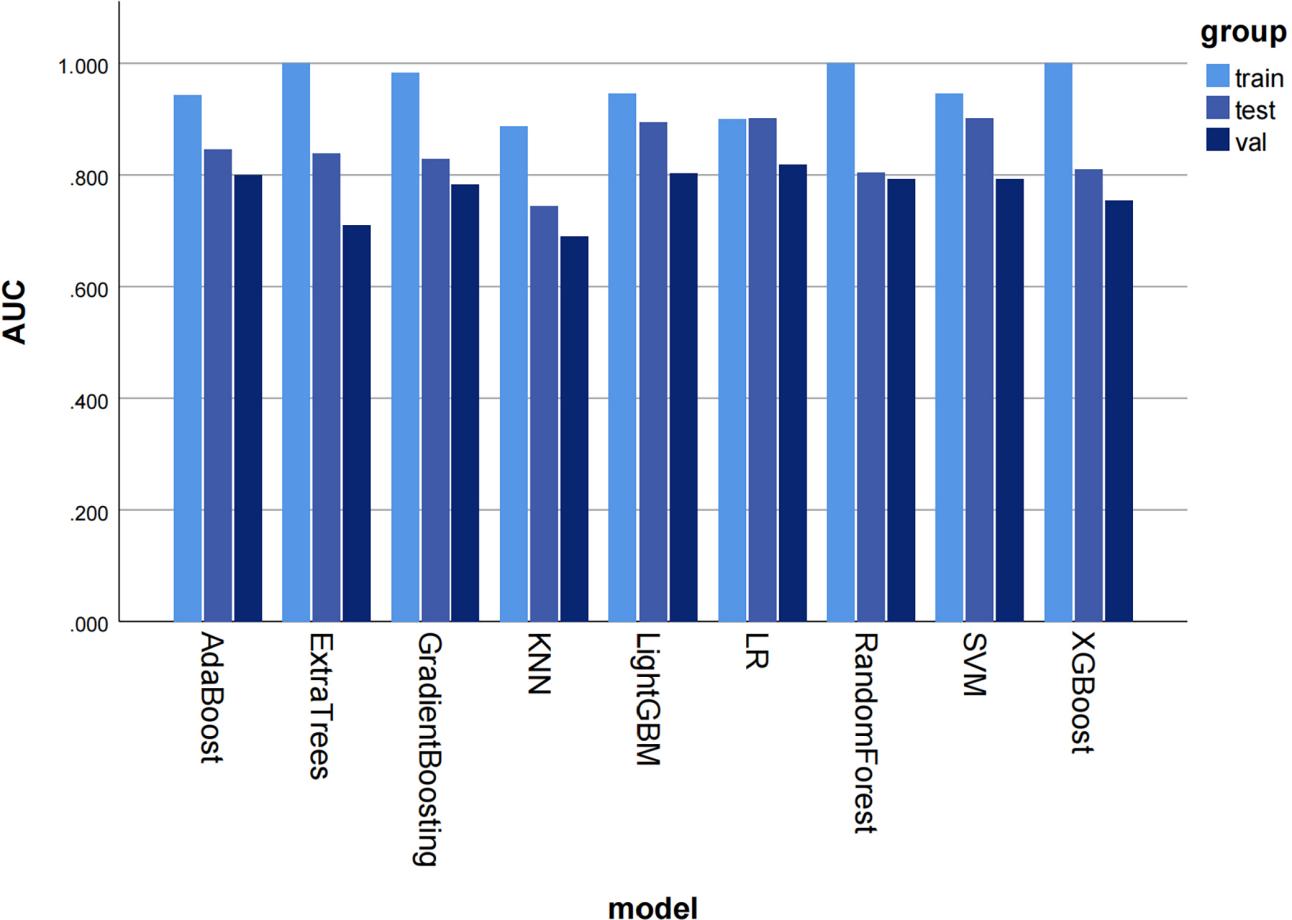
The histogram of AUC values for different machine learning models to diagnose moderate to severe gastric atrophy in training, test, and external validation sets. The AUC represents the area under the ROC curve generated by the classifier based on different decision thresholds to evaluate the model performance. Overall, a model’s performance improves as the AUC approaches 1. AUC, area under the curve; ROC, receiver operator characteristic curve; KNN, K Nearest Neighbor; LR, logistic regression; SVM, Support Vector Machine.

**Figure 4:**
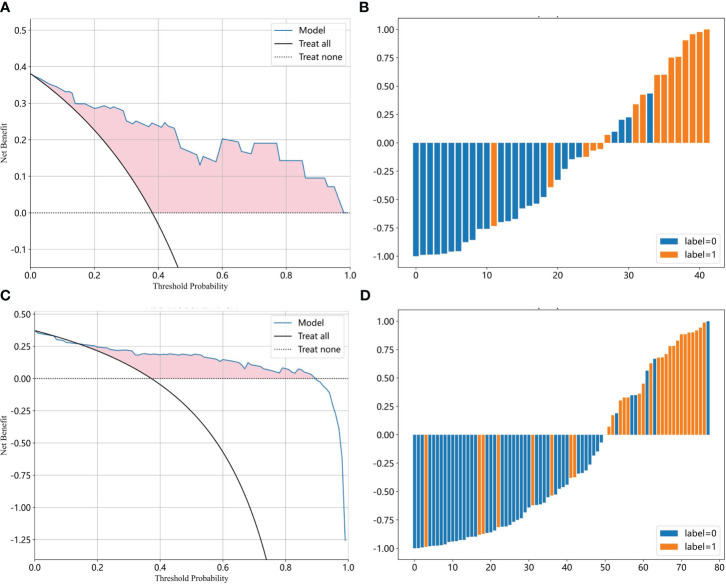
The efficacy of the LR model for moderate to severe gastric atrophy. **(A, B)** The DCA and prediction histogram in the test set. **(C, D)** The DCA and prediction histogram in the external validation set. The DCA is used to evaluate the net benefit of the model under different decision thresholds. We often draw a reference line, a baseline for making random decisions without any predictions. If the net benefit curve of the model is above the baseline, it indicates that the model has better clinical utility relative to the baseline. Prediction histograms can display and analyze the distribution of prediction results, which helps us evaluate the model performance. LR, logistic Regression; DCA, decision curve analysis.

Most ML algorithms exhibited relatively satisfied diagnostic performance in the training set (AUC: 0.887-1.000), test set (AUC: 0.744-0.901), and external validation set (AUC: 0.689-0.818). The LR model achieved the highest AUC value of 0.900 (95%CI: 0.852-0.947) in the training set, 0.901 (95%CI: 0.807- 0.996) in the test set, and 0.818 (95%CI: 0.714-0.923) in the validation set. **Figure 3** and **Table 1** show the detailed performance of various models. We further compared our model with previously reported models (10–13), which is shown in **Table 2**. In addition, histograms and DCA showed good calibration and clinical benefit of the LR model (**Figure 4**).

**Table 1:** Efficacy of machine learning in diagnosing moderate to severe gastric atrophy.

Model	Accuracy	AUC	95% CI	Sensitivity	Specificity	PPV	NPV	Threshold	Group
LR	0.852	0.900	0.852 - 0.947	0.781	0.895	0.820	0.870	0.470	train
	0.857	0.901	0.807 - 0.996	0.875	0.846	0.778	0.917	0.428	test
	0.821	0.818	0.714 - 0.920	0.724	0.896	0.778	0.843	0.536	val
SVM	0.905	0.945	0.909 - 0.982	0.891	0.914	0.864	0.932	0.409	train
	0.881	0.901	0.807 - 0.996	0.750	0.962	0.923	0.862	0.572	test
	0.731	0.792	0.681 - 0.902	0.828	0.673	0.600	0.868	0.357	val
KNN	0.811	0.887	0.841 - 0.933	0.875	0.771	0.700	0.910	0.400	train
	0.667	0.744	0.584 - 0.904	0.750	0.615	0.545	0.800	0.400	test
	0.731	0.689	0.557 - 0.820	0.552	0.837	0.667	0.759	0.600	val
RF	0.994	0.999	0.998 - 1.000	1.000	0.990	0.985	1.000	0.500	train
	0.857	0.804	0.641 - 0.967	0.625	1.000	1.000	0.812	0.700	test
	0.782	0.793	0.678 - 0.908	0.655	0.857	0.731	0.808	0.600	val
ET	1.000	1.000	1.000 - 1.000	1.000	1.000	1.000	1.000	1.000	train
	0.786	0.838	0.707 - 0.968	0.812	0.769	0.684	0.870	0.500	test
	0.756	0.709	0.580 - 0.838	0.655	0.833	0.679	0.800	0.500	val
XGB	0.994	1.000	0.999 - 1.000	1.000	0.990	0.985	1.000	0.475	train
	0.762	0.810	0.667 - 0.953	0.812	0.731	0.650	0.864	0.364	test
	0.744	0.753	0.637 - 0.868	0.724	0.755	0.636	0.822	0.359	val
LGBM	0.882	0.945	0.915 - 0.975	0.875	0.886	0.824	0.921	0.411	train
	0.833	0.894	0.791 - 0.997	0.875	0.808	0.737	0.913	0.349	test
	0.795	0.803	0.695 - 0.910	0.690	0.875	0.741	0.824	0.434	val
GB	0.929	0.983	0.970 - 0.997	0.984	0.895	0.851	0.989	0.300	train
	0.810	0.828	0.699 - 0.958	0.750	0.846	0.750	0.846	0.429	test
	0.782	0.782	0.675 - 0.889	0.517	0.939	0.833	0.767	0.507	val
AB	0.882	0.942	0.910 - 0.974	0.766	0.952	0.907	0.870	0.506	train
	0.833	0.85	0.712 0.978	0.562	1.000	1.000	0.788	0.563	test
	0.795	0.800	0.692 - 0.909	0.724	0.837	0.724	0.837	0.497	val

LR, Logistic Regression; SVM, Support Vector Machine; KNN, K Nearest Neighbor; ET, Extra Trees; XGB, Extreme Gradient Boosting; LGBM, Light Gradient Boosting Machine; GB, Gradient Boosting; AB, AdaBoost; AUC, Area Under the Curve; CI, Confidence Interval; PPV, Positive Predictive Value; NPV, Negative Predictive Value.

**Table 2:** The comparison of our model with the previous model.

model	Number of images train/test/val	Algorithm	ML/DL	discrimination	AUC	SE	SP
our model	169/42/78	LR	ML	moderate to severe CAG	0.818	0.724	0.896
Ma B, 2020	534/153/76	Inception v3	DL	whether CG	NR	0.958	NR
Ba W, 2021	1008/100/142	DeepLab v3	DL	whether CAG	0.910	0.952	0.992
Barmpoutis P, 2022	85	GAGL-VTNet	DL	whether CAG	NR	0.940	NR
Fang S, 2023	1745/435/545	GasMIL	DL	grade of CAG	0.877	0.700	0.700

CAG, Chronic Atrophic Gastritis; CG, Chronic Gastritis; LR, Logistic Regression; ML, Machine Learning; DL, Deep Learning; AUC, Area Under the Curve; SE, Sensitivity; SP, Specificity; NR, Not Report.

### 3.4 LR-based PS with GC and endoscopic atrophy grading

We selected 0.47 as the cutoff value according to the Youden index and converted the LR model into a pathology score (PS) for gastric atrophy, including high and low PS. In the univariate logistic regression analysis, the sex(OR=2.56, 95%CI: 1.07-6.15; P=0.035), age(OR=1.04, 95%CI: 1.01-1.08; P=0.025) and PS (OR=5.40, 95%CI: 2.55-11.43; P<0.001) was associated with GC. Subsequently, we conducted a multivariate logistic regression analysis, which adjusted the sex and age. The result showed that PS was an independent risk factor for GC (OR=5.70, 95%CI: 2.63-12.33; P<0.001) (**Table 3**). Compared to the low PS, the high PS would increase the risk of GC by more than five times. Besides, we also explore the correlation between the PS and endoscopic atrophy grading (Kimura Takemoto classification). The result showed that the PS was also associated with the endoscopic atrophy grading (Z=-2.478, P=0.013) (**Supplementary Figure S2**).

**Table 3:** Univariate and multivariate logistic regression analysis of PS and GC.

	Non-GC	GC	Univariate		Multivariate	
			*OR(95%CI)*	*P*	*OR(95%CI)*	*P*
Total	165	41				
Sex, n			2.56(1.07-6.15)	0.035	2.61(1.04-6.54)	0.041
Female	57	7				
Male	108	34				
Age, Mean (SD)	59(10)	64(10)	1.04(1.01-1.08)	0.025	1.04(1.00-1.09)	0.039
Smoking, n			1.40(0.70-2.79)	0.341		
No	102	22				
Yes	63	19				
Alcohol, n			1.26(0.63-2.51)	0.507		
No	82	18				
Yes	83	23				
PS, n			5.40(2.55-11.43)	<0.001	5.70(2.63-12.33)	<0.001
Low	114	12				
High	51	29				

n, number; GC, Gastric Cancer; OR, Odd Ratio; CI, Conference Interval; SD, Standard Deviation; PS, Pathology Score for Gastric Atrophy.

## 4 Discussion

The accurate diagnosis of atrophy is vital in clinical practice for two main reasons. Firstly, atrophy is considered a precancerous condition, and its early identification and follow-up are crucial for early diagnosis and treatment of GC (14, 15). However, mild and focal atrophy usually does not progress to GC in the short term, so we should pay more attention to patients with moderate to severe atrophy to reduce unnecessary anxiety of patients. Pathologists diagnose the degree of atrophy mainly based on the reduction of gastric intrinsic glands using VAS (16). This method has relatively high requirements for biopsies, with low intra- and interobserver agreement.

In recent years, artificial intelligence (AI) has rapidly developed in biomedicine and plays a vital role in the diagnosis, staging, and prognosis of many diseases by integrating medical imaging, such as endoscopic examinations, radiographic images, and pathology (17). ML has been popular for analyzing pathological images (18). In general, there are two main types of feature extraction for pathological analysis: automatic feature extraction based on deep neural networks and manual feature extraction (19). Deep learning algorithms can directly input images for learning without manual feature extraction. They usually focus on fine-tuning parameters to maximize accuracy while minimizing processing time (20). In addition, we could apply the deep neural network-based methods trained on specific disease subtypes to others. However, deep learning often requires larger datasets with difficulty to interpret, which limits their clinical application. Therefore, manual feature extraction methods are more likely to be used for higher-level decision-making tasks, such as disease diagnosis and prognosis prediction, while deep neural network approaches may be more suitable for some low-level tasks, such as object detection or segmentation (21, 22). Considering the sample size and application scenario, we chose the manual feature extraction approach.

There is no consensus on extracting pathological features (6, 19). CellProfiler is free and open-source software that can automatically measure phenotypes in biological images easily and repeatably. It is currently used for digital pathology analysis, allowing clinicians to extract quantitative pathological features with satisfactory performance (8, 23). We used this software to extract quantitative pathological features and subsequently construct pathological scores based on the LR model for gastric atrophy.

Traditionally, the pathological diagnostic indicators of chronic atrophic gastritis mainly include reduced intrinsic glands, intestinal epithelial metaplasia, pseudopyloric gland metaplasia, lymphoid follicular formation, and fibrous tissue hyperplasia. However, we explored some other features that may also related to the diagnosis of the atrophy in our study. In the initial phase of the study, the association of features with atrophy was uncertain, and we selected all features using statistical methods. The result demonstrated that the nuclei shape, texture, and color distribution might be related to the diagnosis of atrophy. For example, the Mean_IdentifyPrimaryObjects_AreaShape_CentralMoment_0_3 and Mean_IdentifyPrimaryObjects_AreaShape_CentralMoment_1_0 described the shape distribution of cell nuclei, Mean_IdentifyPrimaryObjects_AreaShape_HuMoment_6 described the cell nucleus symmetry and smoothness of the shape, Mean_IdentifyPrimaryObjects_AreaShape_Zernike_4_2 and Mean_IdentifyPrimaryObjects_AreaShape_Zernike_6_4 described the symmetry of the cell nuclear outline shape and the extent of unfold, Mean_IdentifyPrimaryObjects_Granularity_4_Hematoxylin, Mean_IdentifyPrimaryObjects_Granularity_6_Hematoxylin, and Mean_IdentifyPrimaryObjects_Texture_DifferenceVariance_Hematoxylin_3_03_256 described the granularity and texture of the cell nucleus. However, our study was just exploratory research, and it is necessary to validate it in the future.

There were also some other artificial intelligence models for the diagnosis of gastritis. For example, Ma B et al (10) constructed a convolutional neural network (CNN)-based system to identify normal mucosa, chronic gastritis, and intestinal-type GC in pathological slides. Ba W et al (13) develop a DeepLab v3 algorithm for the pathological classification of chronic gastritis, including chronic superficial gastritis (CSuG), chronic active gastritis (CAcG), and chronic atrophic gastritis (CAtG). Barmpoutis P et al (12) proposed a digital pathology end-to-end workflow for gastric gland segmentation and classification of gastric atrophy and gastric intestinal metaplasia. Although they have achieved excellent performance, they only identified whether it was atrophic gastritis instead of the grade of gastric atrophy. Later, Fang S et al (11) established a diagnostic approach for gastric biopsy specimens using deep learning, which could also discriminate the grade of the atrophy. However, they all employed deep learning and cannot be explained clearly, which will limit their clinical use.

Our model extracted some features of the images and cell nucleus, which were further used to construct the diagnostic model by machine learning. The model not only has favorable diagnostic efficacy which can improve the consistency of atrophy diagnosis, but it could also explain why the model make the decision. We think our model could be used in many scenarios in the future. Firstly, the number of patients with atrophic gastritis is increasing and the number of professional pathologists is decreasing, so it is urgent to develop an accurate AI model for the automatic diagnosis of gastric atrophy to reduce the burden on pathologists. Our model has favorable performance, which can be integrated into the computer to diagnose gastric atrophy automatically. Especially, the features we extracted were all the local pathological characteristics and it is not required to obtain the whole WSI. Therefore, when the endoscopists cannot obtain the muscularis mucosa, we can consider using this model to judge the degree of atrophy and provide real-time clinical decision support. In addition, with the development of endoscopic techniques, cytoendoscopy will be widely used in the foreseeable future. Our model screened some pathological features related to the degree of gastric atrophy, which may be also suitable for the Cytoendoscopic findings. Finally, the pathological score based on the model is correlated with the endoscopic atrophy grading and GC, so it could also be used to assess the risk of GC and guide the follow-up of patients with atrophic gastritis in the future.

However, our study had several limitations. Firstly, it was a retrospective study, which introduced a potential for selection bias. Secondly, we conducted the study in a single center with a limited sample size, so it is necessary to validate the findings with a more diverse dataset that includes different scanners and institutions. Finally, we may have missed some relevant features when extracting the pathological features. Despite this possibility, it was still meaningful for achieving favorable model performance.

In conclusion, we constructed a diagnostic model associated with endoscopic atrophy grading and GC for gastric atrophy based on pathological characteristics to improve diagnostic consistency.

## Data availability statement

The raw data supporting the conclusions of this article will be made available by the authors, without undue reservation.

## Ethics statement

The studies involving humans were approved by The Ethics Committee of Shandong Provincial Hospital. The studies were conducted in accordance with the local legislation and institutional requirements. Written informed consent for participation was not required from the participants or the participants’ legal guardians/next of kin in accordance with the national legislation and institutional requirements.

## Author contributions

YL: Conceptualization, Data curation, Formal analysis, Methodology, Writing – original draft. BH: Data curation, Formal Analysis, Methodology, Writing – review & editing. TZ: Data curation, Methodology, Writing – review & editing. QX: Data curation, Writing – review & editing. ZL: Data curation, Writing – review & editing. ML: Data curation, Writing – review & editing. YX: Data curation, Writing – review & editing. HX: Conceptualization, Funding acquisition, Supervision, Writing – review & editing.
