# Supplementary material for: Clinical application of machine learning‐based pathomics signature of gastric atrophy
Source: Front Oncol. 2024 Feb 27;14:1289265. doi: 10.3389/fonc.2024.1289265 (PMC10929611; doi:10.3389/fonc.2024.1289265)
Supplement: Supplementary file 1 [file DataSheet_1.docx]

Supplementary Material

**Table S1.** Comprehensive list of quantitative features extracted by the gastric biopsy image processing pipeline

|  | Feature name |
| --- | --- |
| 1 | Correlation_Correlation_Eosin_Hematoxylin |
| 2 | Correlation_Costes_Eosin_Hematoxylin |
| 3 | Correlation_Costes_Hematoxylin_Eosin |
| 4 | Correlation_K_Eosin_Hematoxylin |
| 5 | Correlation_K_Hematoxylin_Eosin |
| 6 | Correlation_Manders_Eosin_Hematoxylin |
| 7 | Correlation_Manders_Hematoxylin_Eosin |
| 8 | Correlation_Overlap_Eosin_Hematoxylin |
| 9 | Correlation_RWC_Eosin_Hematoxylin |
| 10 | Correlation_RWC_Hematoxylin_Eosin |
| 11 | Correlation_Slope_Eosin_Hematoxylin |
| 12 | Count_IdentifyPrimaryObjects |
| 13 | ExecutionTime_03NamesAndTypes |
| 14 | ExecutionTime_05UnmixColors |
| 15 | ExecutionTime_06ColorToGray |
| 16 | ExecutionTime_07IdentifyPrimaryObjects |
| 17 | ExecutionTime_08MeasureImageQuality |
| 18 | ExecutionTime_09MeasureImageIntensity |
| 19 | ExecutionTime_10MeasureColocalization |
| 20 | ExecutionTime_11MeasureGranularity |
| 21 | ExecutionTime_12MeasureTexture |
| 22 | ExecutionTime_13MeasureObjectSizeShape |
| 23 | ExecutionTime_14MeasureObjectIntensity |
| 24 | ExecutionTime_15MeasureObjectIntensityDistribution |
| 25 | Granularity_10_Hematoxylin |
| 26 | Granularity_11_Hematoxylin |
| 27 | Granularity_12_Hematoxylin |
| 28 | Granularity_13_Hematoxylin |
| 29 | Granularity_14_Hematoxylin |
| 30 | Granularity_15_Hematoxylin |
| 31 | Granularity_16_Hematoxylin |
| 32 | Granularity_1_Hematoxylin |
| 33 | Granularity_2_Hematoxylin |
| 34 | Granularity_3_Hematoxylin |
| 35 | Granularity_4_Hematoxylin |
| 36 | Granularity_5_Hematoxylin |
| 37 | Granularity_6_Hematoxylin |
| 38 | Granularity_7_Hematoxylin |
| 39 | Granularity_8_Hematoxylin |
| 40 | Granularity_9_Hematoxylin |
| 41 | ImageQuality_Correlation_Eosin_20 |
| 42 | ImageQuality_Correlation_Hematoxylin_20 |
| 43 | ImageQuality_Correlation_OrigGray_20 |
| 44 | ImageQuality_FocusScore_Eosin |
| 45 | ImageQuality_FocusScore_Hematoxylin |
| 46 | ImageQuality_FocusScore_OrigGray |
| 47 | ImageQuality_LocalFocusScore_Eosin_20 |
| 48 | ImageQuality_LocalFocusScore_Hematoxylin_20 |
| 49 | ImageQuality_LocalFocusScore_OrigGray_20 |
| 50 | ImageQuality_MADIntensity_Eosin |
| 51 | ImageQuality_MADIntensity_Hematoxylin |
| 52 | ImageQuality_MADIntensity_OrigGray |
| 53 | ImageQuality_MaxIntensity_Eosin |
| 54 | ImageQuality_MaxIntensity_Hematoxylin |
| 55 | ImageQuality_MaxIntensity_OrigGray |
| 56 | ImageQuality_MeanIntensity_Eosin |
| 57 | ImageQuality_MeanIntensity_Hematoxylin |
| 58 | ImageQuality_MeanIntensity_OrigGray |
| 59 | ImageQuality_MedianIntensity_Eosin |
| 60 | ImageQuality_MedianIntensity_Hematoxylin |
| 61 | ImageQuality_MedianIntensity_OrigGray |
| 62 | ImageQuality_MinIntensity_OrigGray |
| 63 | ImageQuality_PercentMaximal_Eosin |
| 64 | ImageQuality_PercentMaximal_Hematoxylin |
| 65 | ImageQuality_PercentMaximal_OrigGray |
| 66 | ImageQuality_PercentMinimal_Eosin |
| 67 | ImageQuality_PercentMinimal_Hematoxylin |
| 68 | ImageQuality_PercentMinimal_OrigGray |
| 69 | ImageQuality_PowerLogLogSlope_Eosin |
| 70 | ImageQuality_PowerLogLogSlope_Hematoxylin |
| 71 | ImageQuality_PowerLogLogSlope_OrigGray |
| 72 | ImageQuality_StdIntensity_Eosin |
| 73 | ImageQuality_StdIntensity_Hematoxylin |
| 74 | ImageQuality_StdIntensity_OrigGray |
| 75 | ImageQuality_ThresholdOtsu_Eosin_2W |
| 76 | ImageQuality_ThresholdOtsu_Hematoxylin_2W |
| 77 | ImageQuality_ThresholdOtsu_OrigGray_2W |
| 78 | ImageQuality_TotalIntensity_Eosin |
| 79 | ImageQuality_TotalIntensity_Hematoxylin |
| 80 | ImageQuality_TotalIntensity_OrigGray |
| 81 | Intensity_LowerQuartileIntensity_Eosin |
| 82 | Intensity_LowerQuartileIntensity_Hematoxylin |
| 83 | Intensity_LowerQuartileIntensity_OrigGray |
| 84 | Intensity_MADIntensity_Eosin |
| 85 | Intensity_MADIntensity_Hematoxylin |
| 86 | Intensity_MADIntensity_OrigGray |
| 87 | Intensity_MaxIntensity_Eosin |
| 88 | Intensity_MaxIntensity_Hematoxylin |
| 89 | Intensity_MaxIntensity_OrigGray |
| 90 | Intensity_MeanIntensity_Eosin |
| 91 | Intensity_MeanIntensity_Hematoxylin |
| 92 | Intensity_MeanIntensity_OrigGray |
| 93 | Intensity_MedianIntensity_Eosin |
| 94 | Intensity_MedianIntensity_Hematoxylin |
| 95 | Intensity_MedianIntensity_OrigGray |
| 96 | Intensity_MinIntensity_Eosin |
| 97 | Intensity_MinIntensity_Hematoxylin |
| 98 | Intensity_MinIntensity_OrigGray |
| 99 | Intensity_PercentMaximal_Eosin |
| 100 | Intensity_PercentMaximal_Hematoxylin |
| 101 | Intensity_PercentMaximal_OrigGray |
| 102 | Intensity_Percentile_25_Eosin |
| 103 | Intensity_Percentile_25_Hematoxylin |
| 104 | Intensity_Percentile_25_OrigGray |
| 105 | Intensity_Percentile_75_Eosin |
| 106 | Intensity_Percentile_75_Hematoxylin |
| 107 | Intensity_Percentile_75_OrigGray |
| 108 | Intensity_StdIntensity_Eosin |
| 109 | Intensity_StdIntensity_Hematoxylin |
| 110 | Intensity_StdIntensity_OrigGray |
| 111 | Intensity_TotalIntensity_Eosin |
| 112 | Intensity_TotalIntensity_Hematoxylin |
| 113 | Intensity_TotalIntensity_OrigGray |
| 114 | Intensity_UpperQuartileIntensity_Eosin |
| 115 | Intensity_UpperQuartileIntensity_Hematoxylin |
| 116 | Intensity_UpperQuartileIntensity_OrigGray |
| 117 | Threshold_FinalThreshold_IdentifyPrimaryObjects |
| 118 | Threshold_OrigThreshold_IdentifyPrimaryObjects |
| 119 | Threshold_SumOfEntropies_IdentifyPrimaryObjects |
| 120 | Threshold_WeightedVariance_IdentifyPrimaryObjects |
| 121 | Mean_IdentifyPrimaryObjects_AreaShape_Area |
| 122 | Mean_IdentifyPrimaryObjects_AreaShape_BoundingBoxArea |
| 123 | Mean_IdentifyPrimaryObjects_AreaShape_BoundingBoxMaximum_X |
| 124 | Mean_IdentifyPrimaryObjects_AreaShape_BoundingBoxMaximum_Y |
| 125 | Mean_IdentifyPrimaryObjects_AreaShape_BoundingBoxMinimum_X |
| 126 | Mean_IdentifyPrimaryObjects_AreaShape_BoundingBoxMinimum_Y |
| 127 | Mean_IdentifyPrimaryObjects_AreaShape_Center_X |
| 128 | Mean_IdentifyPrimaryObjects_AreaShape_Center_Y |
| 129 | Mean_IdentifyPrimaryObjects_AreaShape_CentralMoment_0_0 |
| 130 | Mean_IdentifyPrimaryObjects_AreaShape_CentralMoment_0_1 |
| 131 | Mean_IdentifyPrimaryObjects_AreaShape_CentralMoment_0_2 |
| 132 | Mean_IdentifyPrimaryObjects_AreaShape_CentralMoment_0_3 |
| 133 | Mean_IdentifyPrimaryObjects_AreaShape_CentralMoment_1_0 |
| 134 | Mean_IdentifyPrimaryObjects_AreaShape_CentralMoment_1_1 |
| 135 | Mean_IdentifyPrimaryObjects_AreaShape_CentralMoment_1_2 |
| 136 | Mean_IdentifyPrimaryObjects_AreaShape_CentralMoment_1_3 |
| 137 | Mean_IdentifyPrimaryObjects_AreaShape_CentralMoment_2_0 |
| 138 | Mean_IdentifyPrimaryObjects_AreaShape_CentralMoment_2_1 |
| 139 | Mean_IdentifyPrimaryObjects_AreaShape_CentralMoment_2_2 |
| 140 | Mean_IdentifyPrimaryObjects_AreaShape_CentralMoment_2_3 |
| 141 | Mean_IdentifyPrimaryObjects_AreaShape_Compactness |
| 142 | Mean_IdentifyPrimaryObjects_AreaShape_ConvexArea |
| 143 | Mean_IdentifyPrimaryObjects_AreaShape_Eccentricity |
| 144 | Mean_IdentifyPrimaryObjects_AreaShape_EquivalentDiameter |
| 145 | Mean_IdentifyPrimaryObjects_AreaShape_EulerNumber |
| 146 | Mean_IdentifyPrimaryObjects_AreaShape_Extent |
| 147 | Mean_IdentifyPrimaryObjects_AreaShape_FormFactor |
| 148 | Mean_IdentifyPrimaryObjects_AreaShape_HuMoment_0 |
| 149 | Mean_IdentifyPrimaryObjects_AreaShape_HuMoment_1 |
| 150 | Mean_IdentifyPrimaryObjects_AreaShape_HuMoment_2 |
| 151 | Mean_IdentifyPrimaryObjects_AreaShape_HuMoment_3 |
| 152 | Mean_IdentifyPrimaryObjects_AreaShape_HuMoment_4 |
| 153 | Mean_IdentifyPrimaryObjects_AreaShape_HuMoment_5 |
| 154 | Mean_IdentifyPrimaryObjects_AreaShape_HuMoment_6 |
| 155 | Mean_IdentifyPrimaryObjects_AreaShape_InertiaTensorEigenvalues_0 |
| 156 | Mean_IdentifyPrimaryObjects_AreaShape_InertiaTensorEigenvalues_1 |
| 157 | Mean_IdentifyPrimaryObjects_AreaShape_InertiaTensor_0_0 |
| 158 | Mean_IdentifyPrimaryObjects_AreaShape_InertiaTensor_0_1 |
| 159 | Mean_IdentifyPrimaryObjects_AreaShape_InertiaTensor_1_0 |
| 160 | Mean_IdentifyPrimaryObjects_AreaShape_InertiaTensor_1_1 |
| 161 | Mean_IdentifyPrimaryObjects_AreaShape_MajorAxisLength |
| 162 | Mean_IdentifyPrimaryObjects_AreaShape_MaxFeretDiameter |
| 163 | Mean_IdentifyPrimaryObjects_AreaShape_MaximumRadius |
| 164 | Mean_IdentifyPrimaryObjects_AreaShape_MeanRadius |
| 165 | Mean_IdentifyPrimaryObjects_AreaShape_MedianRadius |
| 166 | Mean_IdentifyPrimaryObjects_AreaShape_MinFeretDiameter |
| 167 | Mean_IdentifyPrimaryObjects_AreaShape_MinorAxisLength |
| 168 | Mean_IdentifyPrimaryObjects_AreaShape_NormalizedMoment_0_2 |
| 169 | Mean_IdentifyPrimaryObjects_AreaShape_NormalizedMoment_0_3 |
| 170 | Mean_IdentifyPrimaryObjects_AreaShape_NormalizedMoment_1_1 |
| 171 | Mean_IdentifyPrimaryObjects_AreaShape_NormalizedMoment_1_2 |
| 172 | Mean_IdentifyPrimaryObjects_AreaShape_NormalizedMoment_1_3 |
| 173 | Mean_IdentifyPrimaryObjects_AreaShape_NormalizedMoment_2_0 |
| 174 | Mean_IdentifyPrimaryObjects_AreaShape_NormalizedMoment_2_1 |
| 175 | Mean_IdentifyPrimaryObjects_AreaShape_NormalizedMoment_2_2 |
| 176 | Mean_IdentifyPrimaryObjects_AreaShape_NormalizedMoment_2_3 |
| 177 | Mean_IdentifyPrimaryObjects_AreaShape_NormalizedMoment_3_0 |
| 178 | Mean_IdentifyPrimaryObjects_AreaShape_NormalizedMoment_3_1 |
| 179 | Mean_IdentifyPrimaryObjects_AreaShape_NormalizedMoment_3_2 |
| 180 | Mean_IdentifyPrimaryObjects_AreaShape_NormalizedMoment_3_3 |
| 181 | Mean_IdentifyPrimaryObjects_AreaShape_Orientation |
| 182 | Mean_IdentifyPrimaryObjects_AreaShape_Perimeter |
| 183 | Mean_IdentifyPrimaryObjects_AreaShape_Solidity |
| 184 | Mean_IdentifyPrimaryObjects_AreaShape_SpatialMoment_0_0 |
| 185 | Mean_IdentifyPrimaryObjects_AreaShape_SpatialMoment_0_1 |
| 186 | Mean_IdentifyPrimaryObjects_AreaShape_SpatialMoment_0_2 |
| 187 | Mean_IdentifyPrimaryObjects_AreaShape_SpatialMoment_0_3 |
| 188 | Mean_IdentifyPrimaryObjects_AreaShape_SpatialMoment_1_0 |
| 189 | Mean_IdentifyPrimaryObjects_AreaShape_SpatialMoment_1_1 |
| 190 | Mean_IdentifyPrimaryObjects_AreaShape_SpatialMoment_1_2 |
| 191 | Mean_IdentifyPrimaryObjects_AreaShape_SpatialMoment_1_3 |
| 192 | Mean_IdentifyPrimaryObjects_AreaShape_SpatialMoment_2_0 |
| 193 | Mean_IdentifyPrimaryObjects_AreaShape_SpatialMoment_2_1 |
| 194 | Mean_IdentifyPrimaryObjects_AreaShape_SpatialMoment_2_2 |
| 195 | Mean_IdentifyPrimaryObjects_AreaShape_SpatialMoment_2_3 |
| 196 | Mean_IdentifyPrimaryObjects_AreaShape_Zernike_0_0 |
| 197 | Mean_IdentifyPrimaryObjects_AreaShape_Zernike_1_1 |
| 198 | Mean_IdentifyPrimaryObjects_AreaShape_Zernike_2_0 |
| 199 | Mean_IdentifyPrimaryObjects_AreaShape_Zernike_2_2 |
| 200 | Mean_IdentifyPrimaryObjects_AreaShape_Zernike_3_1 |
| 201 | Mean_IdentifyPrimaryObjects_AreaShape_Zernike_3_3 |
| 202 | Mean_IdentifyPrimaryObjects_AreaShape_Zernike_4_0 |
| 203 | Mean_IdentifyPrimaryObjects_AreaShape_Zernike_4_2 |
| 204 | Mean_IdentifyPrimaryObjects_AreaShape_Zernike_4_4 |
| 205 | Mean_IdentifyPrimaryObjects_AreaShape_Zernike_5_1 |
| 206 | Mean_IdentifyPrimaryObjects_AreaShape_Zernike_5_3 |
| 207 | Mean_IdentifyPrimaryObjects_AreaShape_Zernike_5_5 |
| 208 | Mean_IdentifyPrimaryObjects_AreaShape_Zernike_6_0 |
| 209 | Mean_IdentifyPrimaryObjects_AreaShape_Zernike_6_2 |
| 210 | Mean_IdentifyPrimaryObjects_AreaShape_Zernike_6_4 |
| 211 | Mean_IdentifyPrimaryObjects_AreaShape_Zernike_6_6 |
| 212 | Mean_IdentifyPrimaryObjects_AreaShape_Zernike_7_1 |
| 213 | Mean_IdentifyPrimaryObjects_AreaShape_Zernike_7_3 |
| 214 | Mean_IdentifyPrimaryObjects_AreaShape_Zernike_7_5 |
| 215 | Mean_IdentifyPrimaryObjects_AreaShape_Zernike_7_7 |
| 216 | Mean_IdentifyPrimaryObjects_AreaShape_Zernike_8_0 |
| 217 | Mean_IdentifyPrimaryObjects_AreaShape_Zernike_8_2 |
| 218 | Mean_IdentifyPrimaryObjects_AreaShape_Zernike_8_4 |
| 219 | Mean_IdentifyPrimaryObjects_AreaShape_Zernike_8_6 |
| 220 | Mean_IdentifyPrimaryObjects_AreaShape_Zernike_8_8 |
| 221 | Mean_IdentifyPrimaryObjects_AreaShape_Zernike_9_1 |
| 222 | Mean_IdentifyPrimaryObjects_AreaShape_Zernike_9_3 |
| 223 | Mean_IdentifyPrimaryObjects_AreaShape_Zernike_9_5 |
| 224 | Mean_IdentifyPrimaryObjects_AreaShape_Zernike_9_7 |
| 225 | Mean_IdentifyPrimaryObjects_AreaShape_Zernike_9_9 |
| 226 | Mean_IdentifyPrimaryObjects_Correlation_Correlation_Eosin_Hematoxylin |
| 227 | Mean_IdentifyPrimaryObjects_Correlation_Costes_Eosin_Hematoxylin |
| 228 | Mean_IdentifyPrimaryObjects_Correlation_Costes_Hematoxylin_Eosin |
| 229 | Mean_IdentifyPrimaryObjects_Correlation_K_Eosin_Hematoxylin |
| 230 | Mean_IdentifyPrimaryObjects_Correlation_K_Hematoxylin_Eosin |
| 231 | Mean_IdentifyPrimaryObjects_Correlation_Manders_Eosin_Hematoxylin |
| 232 | Mean_IdentifyPrimaryObjects_Correlation_Manders_Hematoxylin_Eosin |
| 233 | Mean_IdentifyPrimaryObjects_Correlation_Overlap_Eosin_Hematoxylin |
| 234 | Mean_IdentifyPrimaryObjects_Correlation_RWC_Eosin_Hematoxylin |
| 235 | Mean_IdentifyPrimaryObjects_Correlation_RWC_Hematoxylin_Eosin |
| 236 | Mean_IdentifyPrimaryObjects_Granularity_10_Hematoxylin |
| 237 | Mean_IdentifyPrimaryObjects_Granularity_11_Hematoxylin |
| 238 | Mean_IdentifyPrimaryObjects_Granularity_12_Hematoxylin |
| 239 | Mean_IdentifyPrimaryObjects_Granularity_13_Hematoxylin |
| 240 | Mean_IdentifyPrimaryObjects_Granularity_14_Hematoxylin |
| 241 | Mean_IdentifyPrimaryObjects_Granularity_15_Hematoxylin |
| 242 | Mean_IdentifyPrimaryObjects_Granularity_16_Hematoxylin |
| 243 | Mean_IdentifyPrimaryObjects_Granularity_1_Hematoxylin |
| 244 | Mean_IdentifyPrimaryObjects_Granularity_2_Hematoxylin |
| 245 | Mean_IdentifyPrimaryObjects_Granularity_3_Hematoxylin |
| 246 | Mean_IdentifyPrimaryObjects_Granularity_4_Hematoxylin |
| 247 | Mean_IdentifyPrimaryObjects_Granularity_5_Hematoxylin |
| 248 | Mean_IdentifyPrimaryObjects_Granularity_6_Hematoxylin |
| 249 | Mean_IdentifyPrimaryObjects_Granularity_7_Hematoxylin |
| 250 | Mean_IdentifyPrimaryObjects_Granularity_8_Hematoxylin |
| 251 | Mean_IdentifyPrimaryObjects_Granularity_9_Hematoxylin |
| 252 | Mean_IdentifyPrimaryObjects_Intensity_IntegratedIntensityEdge_Eosin |
| 253 | Mean_IdentifyPrimaryObjects_Intensity_IntegratedIntensityEdge_Hematoxylin |
| 254 | Mean_IdentifyPrimaryObjects_Intensity_IntegratedIntensityEdge_OrigGray |
| 255 | Mean_IdentifyPrimaryObjects_Intensity_IntegratedIntensity_Eosin |
| 256 | Mean_IdentifyPrimaryObjects_Intensity_IntegratedIntensity_Hematoxylin |
| 257 | Mean_IdentifyPrimaryObjects_Intensity_IntegratedIntensity_OrigGray |
| 258 | Mean_IdentifyPrimaryObjects_Intensity_LowerQuartileIntensity_Eosin |
| 259 | Mean_IdentifyPrimaryObjects_Intensity_LowerQuartileIntensity_Hematoxylin |
| 260 | Mean_IdentifyPrimaryObjects_Intensity_LowerQuartileIntensity_OrigGray |
| 261 | Mean_IdentifyPrimaryObjects_Intensity_MADIntensity_Eosin |
| 262 | Mean_IdentifyPrimaryObjects_Intensity_MADIntensity_Hematoxylin |
| 263 | Mean_IdentifyPrimaryObjects_Intensity_MADIntensity_OrigGray |
| 264 | Mean_IdentifyPrimaryObjects_Intensity_MassDisplacement_Eosin |
| 265 | Mean_IdentifyPrimaryObjects_Intensity_MassDisplacement_Hematoxylin |
| 266 | Mean_IdentifyPrimaryObjects_Intensity_MassDisplacement_OrigGray |
| 267 | Mean_IdentifyPrimaryObjects_Intensity_MaxIntensityEdge_Eosin |
| 268 | Mean_IdentifyPrimaryObjects_Intensity_MaxIntensityEdge_Hematoxylin |
| 269 | Mean_IdentifyPrimaryObjects_Intensity_MaxIntensityEdge_OrigGray |
| 270 | Mean_IdentifyPrimaryObjects_Intensity_MaxIntensity_Eosin |
| 271 | Mean_IdentifyPrimaryObjects_Intensity_MaxIntensity_Hematoxylin |
| 272 | Mean_IdentifyPrimaryObjects_Intensity_MaxIntensity_OrigGray |
| 273 | Mean_IdentifyPrimaryObjects_Intensity_MeanIntensityEdge_Eosin |
| 274 | Mean_IdentifyPrimaryObjects_Intensity_MeanIntensityEdge_Hematoxylin |
| 275 | Mean_IdentifyPrimaryObjects_Intensity_MeanIntensityEdge_OrigGray |
| 276 | Mean_IdentifyPrimaryObjects_Intensity_MeanIntensity_Eosin |
| 277 | Mean_IdentifyPrimaryObjects_Intensity_MeanIntensity_Hematoxylin |
| 278 | Mean_IdentifyPrimaryObjects_Intensity_MeanIntensity_OrigGray |
| 279 | Mean_IdentifyPrimaryObjects_Intensity_MedianIntensity_Eosin |
| 280 | Mean_IdentifyPrimaryObjects_Intensity_MedianIntensity_Hematoxylin |
| 281 | Mean_IdentifyPrimaryObjects_Intensity_MedianIntensity_OrigGray |
| 282 | Mean_IdentifyPrimaryObjects_Intensity_MinIntensityEdge_Eosin |
| 283 | Mean_IdentifyPrimaryObjects_Intensity_MinIntensityEdge_Hematoxylin |
| 284 | Mean_IdentifyPrimaryObjects_Intensity_MinIntensityEdge_OrigGray |
| 285 | Mean_IdentifyPrimaryObjects_Intensity_MinIntensity_Eosin |
| 286 | Mean_IdentifyPrimaryObjects_Intensity_MinIntensity_Hematoxylin |
| 287 | Mean_IdentifyPrimaryObjects_Intensity_MinIntensity_OrigGray |
| 288 | Mean_IdentifyPrimaryObjects_Intensity_StdIntensityEdge_Eosin |
| 289 | Mean_IdentifyPrimaryObjects_Intensity_StdIntensityEdge_Hematoxylin |
| 290 | Mean_IdentifyPrimaryObjects_Intensity_StdIntensityEdge_OrigGray |
| 291 | Mean_IdentifyPrimaryObjects_Intensity_StdIntensity_Eosin |
| 292 | Mean_IdentifyPrimaryObjects_Intensity_StdIntensity_Hematoxylin |
| 293 | Mean_IdentifyPrimaryObjects_Intensity_StdIntensity_OrigGray |
| 294 | Mean_IdentifyPrimaryObjects_Intensity_UpperQuartileIntensity_Eosin |
| 295 | Mean_IdentifyPrimaryObjects_Intensity_UpperQuartileIntensity_Hematoxylin |
| 296 | Mean_IdentifyPrimaryObjects_Intensity_UpperQuartileIntensity_OrigGray |
| 297 | Mean_IdentifyPrimaryObjects_Location_CenterMassIntensity_X_Eosin |
| 298 | Mean_IdentifyPrimaryObjects_Location_CenterMassIntensity_X_Hematoxylin |
| 299 | Mean_IdentifyPrimaryObjects_Location_CenterMassIntensity_X_OrigGray |
| 300 | Mean_IdentifyPrimaryObjects_Location_CenterMassIntensity_Y_Eosin |
| 301 | Mean_IdentifyPrimaryObjects_Location_CenterMassIntensity_Y_Hematoxylin |
| 302 | Mean_IdentifyPrimaryObjects_Location_CenterMassIntensity_Y_OrigGray |
| 303 | Mean_IdentifyPrimaryObjects_Location_Center_X |
| 304 | Mean_IdentifyPrimaryObjects_Location_Center_Y |
| 305 | Mean_IdentifyPrimaryObjects_Location_MaxIntensity_X_Eosin |
| 306 | Mean_IdentifyPrimaryObjects_Location_MaxIntensity_X_Hematoxylin |
| 307 | Mean_IdentifyPrimaryObjects_Location_MaxIntensity_X_OrigGray |
| 308 | Mean_IdentifyPrimaryObjects_Location_MaxIntensity_Y_Eosin |
| 309 | Mean_IdentifyPrimaryObjects_Location_MaxIntensity_Y_Hematoxylin |
| 310 | Mean_IdentifyPrimaryObjects_Location_MaxIntensity_Y_OrigGray |
| 311 | Mean_IdentifyPrimaryObjects_RadialDistribution_FracAtD_Hematoxylin_1of4 |
| 312 | Mean_IdentifyPrimaryObjects_RadialDistribution_FracAtD_Hematoxylin_2of4 |
| 313 | Mean_IdentifyPrimaryObjects_RadialDistribution_FracAtD_Hematoxylin_3of4 |
| 314 | Mean_IdentifyPrimaryObjects_RadialDistribution_FracAtD_Hematoxylin_4of4 |
| 315 | Mean_IdentifyPrimaryObjects_RadialDistribution_MeanFrac_Hematoxylin_1of4 |
| 316 | Mean_IdentifyPrimaryObjects_RadialDistribution_MeanFrac_Hematoxylin_2of4 |
| 317 | Mean_IdentifyPrimaryObjects_RadialDistribution_MeanFrac_Hematoxylin_3of4 |
| 318 | Mean_IdentifyPrimaryObjects_RadialDistribution_MeanFrac_Hematoxylin_4of4 |
| 319 | Mean_IdentifyPrimaryObjects_RadialDistribution_RadialCV_Hematoxylin_1of4 |
| 320 | Mean_IdentifyPrimaryObjects_RadialDistribution_RadialCV_Hematoxylin_2of4 |
| 321 | Mean_IdentifyPrimaryObjects_RadialDistribution_RadialCV_Hematoxylin_3of4 |
| 322 | Mean_IdentifyPrimaryObjects_RadialDistribution_RadialCV_Hematoxylin_4of4 |
| 323 | Mean_IdentifyPrimaryObjects_RadialDistribution_ZernikeMagnitude_Hematoxylin_0_0 |
| 324 | Mean_IdentifyPrimaryObjects_RadialDistribution_ZernikeMagnitude_Hematoxylin_1_1 |
| 325 | Mean_IdentifyPrimaryObjects_RadialDistribution_ZernikeMagnitude_Hematoxylin_2_0 |
| 326 | Mean_IdentifyPrimaryObjects_RadialDistribution_ZernikeMagnitude_Hematoxylin_2_2 |
| 327 | Mean_IdentifyPrimaryObjects_RadialDistribution_ZernikeMagnitude_Hematoxylin_3_1 |
| 328 | Mean_IdentifyPrimaryObjects_RadialDistribution_ZernikeMagnitude_Hematoxylin_3_3 |
| 329 | Mean_IdentifyPrimaryObjects_RadialDistribution_ZernikeMagnitude_Hematoxylin_4_0 |
| 330 | Mean_IdentifyPrimaryObjects_RadialDistribution_ZernikeMagnitude_Hematoxylin_4_2 |
| 331 | Mean_IdentifyPrimaryObjects_RadialDistribution_ZernikeMagnitude_Hematoxylin_4_4 |
| 332 | Mean_IdentifyPrimaryObjects_RadialDistribution_ZernikeMagnitude_Hematoxylin_5_1 |
| 333 | Mean_IdentifyPrimaryObjects_RadialDistribution_ZernikeMagnitude_Hematoxylin_5_3 |
| 334 | Mean_IdentifyPrimaryObjects_RadialDistribution_ZernikeMagnitude_Hematoxylin_5_5 |
| 335 | Mean_IdentifyPrimaryObjects_RadialDistribution_ZernikeMagnitude_Hematoxylin_6_0 |
| 336 | Mean_IdentifyPrimaryObjects_RadialDistribution_ZernikeMagnitude_Hematoxylin_6_2 |
| 337 | Mean_IdentifyPrimaryObjects_RadialDistribution_ZernikeMagnitude_Hematoxylin_6_4 |
| 338 | Mean_IdentifyPrimaryObjects_RadialDistribution_ZernikeMagnitude_Hematoxylin_6_6 |
| 339 | Mean_IdentifyPrimaryObjects_RadialDistribution_ZernikeMagnitude_Hematoxylin_7_1 |
| 340 | Mean_IdentifyPrimaryObjects_RadialDistribution_ZernikeMagnitude_Hematoxylin_7_3 |
| 341 | Mean_IdentifyPrimaryObjects_RadialDistribution_ZernikeMagnitude_Hematoxylin_7_5 |
| 342 | Mean_IdentifyPrimaryObjects_RadialDistribution_ZernikeMagnitude_Hematoxylin_7_7 |
| 343 | Mean_IdentifyPrimaryObjects_RadialDistribution_ZernikeMagnitude_Hematoxylin_8_0 |
| 344 | Mean_IdentifyPrimaryObjects_RadialDistribution_ZernikeMagnitude_Hematoxylin_8_2 |
| 345 | Mean_IdentifyPrimaryObjects_RadialDistribution_ZernikeMagnitude_Hematoxylin_8_4 |
| 346 | Mean_IdentifyPrimaryObjects_RadialDistribution_ZernikeMagnitude_Hematoxylin_8_6 |
| 347 | Mean_IdentifyPrimaryObjects_RadialDistribution_ZernikeMagnitude_Hematoxylin_8_8 |
| 348 | Mean_IdentifyPrimaryObjects_RadialDistribution_ZernikeMagnitude_Hematoxylin_9_1 |
| 349 | Mean_IdentifyPrimaryObjects_RadialDistribution_ZernikeMagnitude_Hematoxylin_9_3 |
| 350 | Mean_IdentifyPrimaryObjects_RadialDistribution_ZernikeMagnitude_Hematoxylin_9_5 |
| 351 | Mean_IdentifyPrimaryObjects_RadialDistribution_ZernikeMagnitude_Hematoxylin_9_7 |
| 352 | Mean_IdentifyPrimaryObjects_RadialDistribution_ZernikeMagnitude_Hematoxylin_9_9 |
| 353 | Mean_IdentifyPrimaryObjects_RadialDistribution_ZernikePhase_Hematoxylin_0_0 |
| 354 | Mean_IdentifyPrimaryObjects_RadialDistribution_ZernikePhase_Hematoxylin_1_1 |
| 355 | Mean_IdentifyPrimaryObjects_RadialDistribution_ZernikePhase_Hematoxylin_2_0 |
| 356 | Mean_IdentifyPrimaryObjects_RadialDistribution_ZernikePhase_Hematoxylin_2_2 |
| 357 | Mean_IdentifyPrimaryObjects_RadialDistribution_ZernikePhase_Hematoxylin_3_1 |
| 358 | Mean_IdentifyPrimaryObjects_RadialDistribution_ZernikePhase_Hematoxylin_3_3 |
| 359 | Mean_IdentifyPrimaryObjects_RadialDistribution_ZernikePhase_Hematoxylin_4_0 |
| 360 | Mean_IdentifyPrimaryObjects_RadialDistribution_ZernikePhase_Hematoxylin_4_2 |
| 361 | Mean_IdentifyPrimaryObjects_RadialDistribution_ZernikePhase_Hematoxylin_4_4 |
| 362 | Mean_IdentifyPrimaryObjects_RadialDistribution_ZernikePhase_Hematoxylin_5_1 |
| 363 | Mean_IdentifyPrimaryObjects_RadialDistribution_ZernikePhase_Hematoxylin_5_3 |
| 364 | Mean_IdentifyPrimaryObjects_RadialDistribution_ZernikePhase_Hematoxylin_5_5 |
| 365 | Mean_IdentifyPrimaryObjects_RadialDistribution_ZernikePhase_Hematoxylin_6_0 |
| 366 | Mean_IdentifyPrimaryObjects_RadialDistribution_ZernikePhase_Hematoxylin_6_2 |
| 367 | Mean_IdentifyPrimaryObjects_RadialDistribution_ZernikePhase_Hematoxylin_6_4 |
| 368 | Mean_IdentifyPrimaryObjects_RadialDistribution_ZernikePhase_Hematoxylin_6_6 |
| 369 | Mean_IdentifyPrimaryObjects_RadialDistribution_ZernikePhase_Hematoxylin_7_1 |
| 370 | Mean_IdentifyPrimaryObjects_RadialDistribution_ZernikePhase_Hematoxylin_7_3 |
| 371 | Mean_IdentifyPrimaryObjects_RadialDistribution_ZernikePhase_Hematoxylin_7_5 |
| 372 | Mean_IdentifyPrimaryObjects_RadialDistribution_ZernikePhase_Hematoxylin_7_7 |
| 373 | Mean_IdentifyPrimaryObjects_RadialDistribution_ZernikePhase_Hematoxylin_8_0 |
| 374 | Mean_IdentifyPrimaryObjects_RadialDistribution_ZernikePhase_Hematoxylin_8_2 |
| 375 | Mean_IdentifyPrimaryObjects_RadialDistribution_ZernikePhase_Hematoxylin_8_4 |
| 376 | Mean_IdentifyPrimaryObjects_RadialDistribution_ZernikePhase_Hematoxylin_8_6 |
| 377 | Mean_IdentifyPrimaryObjects_RadialDistribution_ZernikePhase_Hematoxylin_8_8 |
| 378 | Mean_IdentifyPrimaryObjects_RadialDistribution_ZernikePhase_Hematoxylin_9_1 |
| 379 | Mean_IdentifyPrimaryObjects_RadialDistribution_ZernikePhase_Hematoxylin_9_3 |
| 380 | Mean_IdentifyPrimaryObjects_RadialDistribution_ZernikePhase_Hematoxylin_9_5 |
| 381 | Mean_IdentifyPrimaryObjects_RadialDistribution_ZernikePhase_Hematoxylin_9_7 |
| 382 | Mean_IdentifyPrimaryObjects_RadialDistribution_ZernikePhase_Hematoxylin_9_9 |
| 383 | Mean_IdentifyPrimaryObjects_Texture_AngularSecondMoment_Hematoxylin_3_00_256 |
| 384 | Mean_IdentifyPrimaryObjects_Texture_AngularSecondMoment_Hematoxylin_3_01_256 |
| 385 | Mean_IdentifyPrimaryObjects_Texture_AngularSecondMoment_Hematoxylin_3_02_256 |
| 386 | Mean_IdentifyPrimaryObjects_Texture_AngularSecondMoment_Hematoxylin_3_03_256 |
| 387 | Mean_IdentifyPrimaryObjects_Texture_Contrast_Hematoxylin_3_00_256 |
| 388 | Mean_IdentifyPrimaryObjects_Texture_Contrast_Hematoxylin_3_01_256 |
| 389 | Mean_IdentifyPrimaryObjects_Texture_Contrast_Hematoxylin_3_02_256 |
| 390 | Mean_IdentifyPrimaryObjects_Texture_Contrast_Hematoxylin_3_03_256 |
| 391 | Mean_IdentifyPrimaryObjects_Texture_Correlation_Hematoxylin_3_00_256 |
| 392 | Mean_IdentifyPrimaryObjects_Texture_Correlation_Hematoxylin_3_01_256 |
| 393 | Mean_IdentifyPrimaryObjects_Texture_Correlation_Hematoxylin_3_02_256 |
| 394 | Mean_IdentifyPrimaryObjects_Texture_Correlation_Hematoxylin_3_03_256 |
| 395 | Mean_IdentifyPrimaryObjects_Texture_DifferenceEntropy_Hematoxylin_3_00_256 |
| 396 | Mean_IdentifyPrimaryObjects_Texture_DifferenceEntropy_Hematoxylin_3_01_256 |
| 397 | Mean_IdentifyPrimaryObjects_Texture_DifferenceEntropy_Hematoxylin_3_02_256 |
| 398 | Mean_IdentifyPrimaryObjects_Texture_DifferenceEntropy_Hematoxylin_3_03_256 |
| 399 | Mean_IdentifyPrimaryObjects_Texture_DifferenceVariance_Hematoxylin_3_00_256 |
| 400 | Mean_IdentifyPrimaryObjects_Texture_DifferenceVariance_Hematoxylin_3_01_256 |
| 401 | Mean_IdentifyPrimaryObjects_Texture_DifferenceVariance_Hematoxylin_3_02_256 |
| 402 | Mean_IdentifyPrimaryObjects_Texture_DifferenceVariance_Hematoxylin_3_03_256 |
| 403 | Mean_IdentifyPrimaryObjects_Texture_Entropy_Hematoxylin_3_00_256 |
| 404 | Mean_IdentifyPrimaryObjects_Texture_Entropy_Hematoxylin_3_01_256 |
| 405 | Mean_IdentifyPrimaryObjects_Texture_Entropy_Hematoxylin_3_02_256 |
| 406 | Mean_IdentifyPrimaryObjects_Texture_Entropy_Hematoxylin_3_03_256 |
| 407 | Mean_IdentifyPrimaryObjects_Texture_InfoMeas1_Hematoxylin_3_00_256 |
| 408 | Mean_IdentifyPrimaryObjects_Texture_InfoMeas1_Hematoxylin_3_01_256 |
| 409 | Mean_IdentifyPrimaryObjects_Texture_InfoMeas1_Hematoxylin_3_02_256 |
| 410 | Mean_IdentifyPrimaryObjects_Texture_InfoMeas1_Hematoxylin_3_03_256 |
| 411 | Mean_IdentifyPrimaryObjects_Texture_InfoMeas2_Hematoxylin_3_00_256 |
| 412 | Mean_IdentifyPrimaryObjects_Texture_InfoMeas2_Hematoxylin_3_01_256 |
| 413 | Mean_IdentifyPrimaryObjects_Texture_InfoMeas2_Hematoxylin_3_02_256 |
| 414 | Mean_IdentifyPrimaryObjects_Texture_InfoMeas2_Hematoxylin_3_03_256 |
| 415 | Mean_IdentifyPrimaryObjects_Texture_InverseDifferenceMoment_Hematoxylin_3_00_256 |
| 416 | Mean_IdentifyPrimaryObjects_Texture_InverseDifferenceMoment_Hematoxylin_3_01_256 |
| 417 | Mean_IdentifyPrimaryObjects_Texture_InverseDifferenceMoment_Hematoxylin_3_02_256 |
| 418 | Mean_IdentifyPrimaryObjects_Texture_InverseDifferenceMoment_Hematoxylin_3_03_256 |
| 419 | Mean_IdentifyPrimaryObjects_Texture_SumAverage_Hematoxylin_3_00_256 |
| 420 | Mean_IdentifyPrimaryObjects_Texture_SumAverage_Hematoxylin_3_01_256 |
| 421 | Mean_IdentifyPrimaryObjects_Texture_SumAverage_Hematoxylin_3_02_256 |
| 422 | Mean_IdentifyPrimaryObjects_Texture_SumAverage_Hematoxylin_3_03_256 |
| 423 | Mean_IdentifyPrimaryObjects_Texture_SumEntropy_Hematoxylin_3_00_256 |
| 424 | Mean_IdentifyPrimaryObjects_Texture_SumEntropy_Hematoxylin_3_01_256 |
| 425 | Mean_IdentifyPrimaryObjects_Texture_SumEntropy_Hematoxylin_3_02_256 |
| 426 | Mean_IdentifyPrimaryObjects_Texture_SumEntropy_Hematoxylin_3_03_256 |
| 427 | Mean_IdentifyPrimaryObjects_Texture_SumVariance_Hematoxylin_3_00_256 |
| 428 | Mean_IdentifyPrimaryObjects_Texture_SumVariance_Hematoxylin_3_01_256 |
| 429 | Mean_IdentifyPrimaryObjects_Texture_SumVariance_Hematoxylin_3_02_256 |
| 430 | Mean_IdentifyPrimaryObjects_Texture_SumVariance_Hematoxylin_3_03_256 |
| 431 | Mean_IdentifyPrimaryObjects_Texture_Variance_Hematoxylin_3_00_256 |
| 432 | Mean_IdentifyPrimaryObjects_Texture_Variance_Hematoxylin_3_01_256 |
| 433 | Mean_IdentifyPrimaryObjects_Texture_Variance_Hematoxylin_3_02_256 |
| 434 | Mean_IdentifyPrimaryObjects_Texture_Variance_Hematoxylin_3_03_256 |


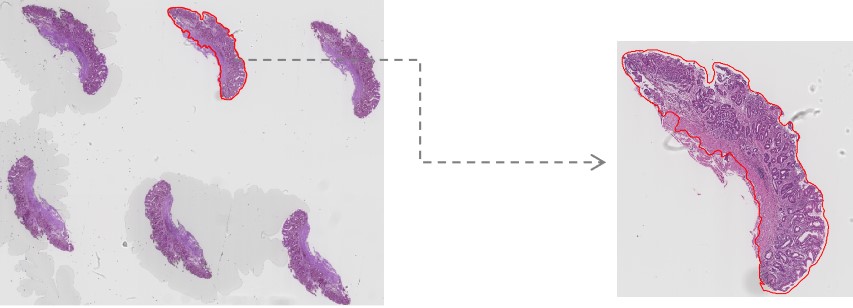


**Figure.S1** The selection of the representative areas with best staining by Qupath software.


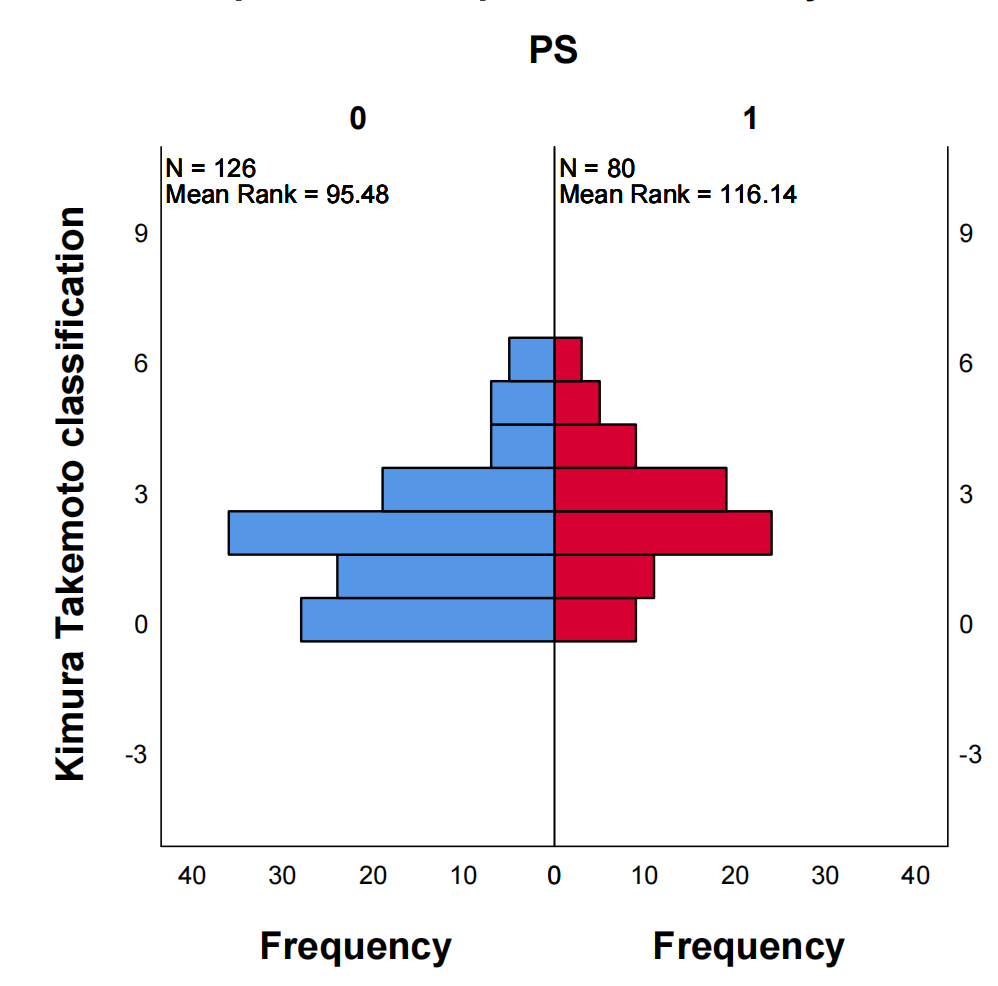


**Figure.S2** The correlation between PS and endoscopic atrophy grading (Kimura Takemoto classification). PS : Pathological Score for Gastric Atrophy.
